# Genetic Predisposition to Schizophrenia and Depressive Disorder Comorbidity

**DOI:** 10.3390/genes13030457

**Published:** 2022-03-02

**Authors:** Natalia A. Shnayder, Maxim A. Novitsky, Nikolay G. Neznanov, Oleg V. Limankin, Azat R. Asadullin, Artem V. Petrov, Diana V. Dmitrenko, Ekaterina A. Narodova, Natalia V. Popenko, Regina F. Nasyrova

**Affiliations:** 1 Institute of Personalized Psychiatry and Neurology, Shared Core Facilities, V.M. Bekhterev National Medical Research Centre for Psychiatry and Neurology, 192019 Saint Petersburg, Russia; maximnovitsky93@gmail.com (M.A.N.); nezn@bekhterev.ru (N.G.N.); 2 Shared Core Facilities “Molecular and Cell Technologies”, V.F. Voino-Yasenetsky Krasnoyarsk State Medical University, 660022 Krasnoyarsk, Russia; artvpetrov@mail.ru (A.V.P.); mart2802@yandex.ru (D.V.D.); katya_n2001@mail.ru (E.A.N.); popenkonv@yandex.ru (N.V.P.); 3 Department of Psychiatry and Addiction, I.M. Pavlov First Saint Petersburg State Medical University, 197022 Saint Petersburg, Russia; 4 P.P. Kashchenko Saint Petersburg Psychiatric Hospital No. 1, Gatchinsky District, 190005 Leningrad, Russia; limankin@mail.ru; 5 Department of Psychotherapy and Sexual Medicine, I.I. Mechnikov North-Western State University, 191015 Saint Petersburg, Russia; 6 Department of Social Psychiatry and Psychology, Saint Petersburg Postgraduate Institute of Medical Experts, 194044 Saint Petersburg, Russia; 7 Department of Psychiatry and Addiction, Bashkir State Medical University, 450008 Ufa, Russia; droar@yandex.ru; 8 International Centre for Education and Research in Neuropsychiatry, Samara State Medical University, 443099 Samara, Russia

**Keywords:** schizophrenia, depression, comorbidity, pathophysiology, genetics, predictor

## Abstract

*Background*: Patients with schizophrenia have an increased risk of depressive disorders compared to the general population. The comorbidity between schizophrenia and depression suggests a potential coincidence of the pathophysiology and/or genetic predictors of these mental disorders. The aim of this study was to review the potential genetic predictors of schizophrenia and depression comorbidity. *Materials and Methods*: We carried out research and analysis of publications in the databases PubMed, Springer, Wiley Online Library, Taylor & Francis Online, Science Direct, and eLIBRARY.RU using keywords and their combinations. The search depth was the last 10 years (2010–2020). Full-text original articles, reviews, meta-analyses, and clinical observations were analyzed. A total of 459 articles were found, of which 45 articles corresponding to the purpose of this study were analyzed in this topic review. *Results*: Overlap in the symptoms and genetic predictors between these disorders suggests that a common etiological mechanism may underlie the presentation of comorbid depression in schizophrenia. The molecular mechanisms linking schizophrenia and depression are polygenic. The most studied candidate genes are *GRIN1*, *GPM6A*, *SEPTIN4*, *TPH1*, *TPH2*, *CACNA1C*, *CACNB2*, and *BCL9.* *Conclusion*: Planning and conducting genome-wide and associative genetic studies of the comorbid conditions under consideration in psychiatry is important for the development of biological and clinical predictors and a personalized therapy strategy for schizophrenia. However, it should be recognized that the problems of predictive and personalized psychiatry in the diagnosis and treatment of schizophrenia and comorbid disorders are far from being resolved.

## 1. Introduction

Schizophrenia and depression are socially significant mental disorders that contribute significantly to the global burden of disease [1,2,3]. On the one hand, patients with schizophrenia have an increased risk of depressive disorders compared to the general population [4]. The development of depression is possible at all stages of the course of schizophrenia, starting with the first psychotic episode [5,6,7,8]. Depressive disorders in patients suffering from schizophrenia are associated with a high risk of suicide [9,10]. Methodological differences in the diagnosis and timescale of assessment of depressive disorders in schizophrenia [11] indicate a wide range of data on the frequency of depressive symptoms reported by patients with schizophrenia [12]. The frequency of occurrence of depressive disorders in patients with schizophrenia is variable and depends on the region of residence and ethnicity. There is a higher incidence of depressive disorders in schizophrenia in the Middle East (72.7%), Russia (61.8%), and residents of Europe (39.6%) compared with residents of the Pacific Ocean region (37.3%) and Southeast Asian countries (32%) [13]. On the other hand, it has been shown that patients with depression have an increased risk of developing psychotic disorders. At the same time, patients with depression may have a high risk of developing schizophrenia [14,15,16,17,18,19]. In addition, the appearance of psychotic symptoms in depression, considered as a separate clinical subtype of depression, called psychotic depression or depression with psychotic symptoms, is associated with increased severity of depressive symptoms [20,21].

This relationship (comorbidity) between schizophrenia and depression suggests a potential coincidence of the pathophysiology and/or etiology of these two mental disorders. However, the comorbidity of psychotic and affective symptoms has been a controversial issue in psychiatry for many years. This is reflected in the discussion about schizoaffective disorder, which currently remains a separate diagnosis characterized by the presence of an episode of severe affective disorder (depressive or manic) that occurs simultaneously with schizophrenia [22]. The low diagnostic reliability of a number of scales and questionnaires for the diagnosis of depression in patients with schizophrenia has led some authors to doubt the inclusion of schizoaffective disorder as a separate pathological condition [23,24]. It remains unclear whether depressive symptoms should be considered as a symptom of schizophrenia, comorbidity, or non-connectedness of epiphenomena [25].

Some negative symptoms of schizophrenia and depressive symptoms partially coincide, for example, anhedonia, abulia, alogia, amotivation and volitional states, and social isolation [26]. This suggested that depressive disorders are part of the schizophrenia syndrome complex [27,28,29,30], which is confirmed by the high prevalence of depressive symptoms in schizophrenia and the link between depression and other manifestations of schizophrenia [12].

As an alternative, depressive disorders in schizophrenia may partially be an adverse reaction of antipsychotics (APs) [31,32], secondary to other concomitant diseases, such as substance abuse or an unclear reaction to the consequences of the disorder [33,34,35,36,37,38].

Regardless of the status of depressive symptoms as major or comorbid with schizophrenia, it is obvious that there is some overlap in the presentation of both disorders.

Along with genetic and environmental factors, genetic and epigenetic factors play an important role in the pathogenesis of affective disorders [39,40]. There is increasing evidence of common genetic risk factors for both schizophrenia and depression. A genome-wide associative study of five major psychiatric disorders showed that single-nucleotide variants (SNVs) in chromosomal loci 3p21 and 10q24, as well as the *CACNA1C* and *CACNB2* genes encoding calcium channel subunits, are largely associated with schizophrenia, depression, bipolar disorder, attention deficit hyperactivity disorder (ADHD), and autism spectrum disorders (ASDs) [41]. In addition, a subgroup of patients with schizophrenia who also suffer from depression turned out to be useful for searching for genetic associations, including the following candidate genes: the *GRIN1* gene encoding the *N*-methyl-d-aspartate receptor (NMDAR) subunit; the *GPM6A* gene encoding a glycoprotein modulating stress in the hippocampus [42,43,44].

A growing number of associative genetic studies in the last decade point to possible common molecular pathways underlying schizophrenia and depression. Despite the rapid development of fundamental and clinical research in the field of neuroscience and psychiatry, the etiology and pathophysiology of both schizophrenia and depression remain insufficiently studied. However, it is clear that there is some relationship between schizophrenia and depression, which affects the risk and severity of both disorders. Understanding the neurobiology linking schizophrenia and depression can improve our understanding of both disorders and help in a personalized approach to their therapy tactics.

The objective of this narrative review was to research candidate genes associated with schizophrenia and depressive disorder comorbidity.

## 2. Materials and Methods

The search strategy was as follows:

- We used keywords “schizophrenia”, “depression”, “comorbidity”, “pathophysiology”, “mechanism”, “genetics”, and their combinations to search for full-text articles in the PubMed, Springer, Wiley Online Library, Taylor & Francis Online, APA PsycInfo, CORE, Science Direct, and eLIBRARY.RU databases.
- We analyzed associative genetic studies, GWAS, cross-sectional studies, case–control studies, case studies, systematic reviews, meta-analyses, and Cochrane reviews. Articles published from January 2011 to November 2021 were analyzed. The final date of the search was 15 November 2021.
- Analyzed data were preselected from identified studies by the titles and abstracts or from the entire publication if titles and abstracts did not provide sufficient information on the type of study.
  - English and Russian languages were included.
  - Duplicate articles were excluded from the analysis.

In addition, the analysis included more recent publications of historical interest.

A total of 459 articles were found, of which 45 articles corresponding to the purpose of this study were analyzed in this paper.

## 3. Results

**Figure 1:**
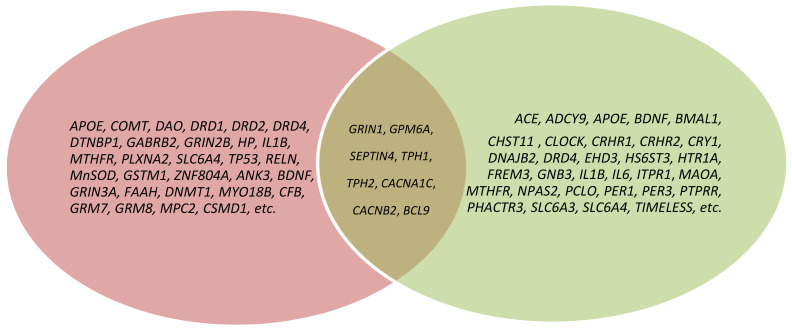
Venn diagram depicting the overlap of genes across schizophrenia and depression; pink color—schizophrenia genes; green color—depression genes; brown color—genes of schizophrenia and depression comorbidity.

As a result of our analysis, we show that the molecular mechanisms linking schizophrenia and depression are polygenic. However, the most studied candidate genes of schizophrenia and depression comorbidity (overlap) are the *GRIN1*, *GPM6A*, *SEPTIN4*, *TPH1*, *TPH2*, *CACNA1C*, *CACNB2*, and *BCL9* genes (Figure 1, Table 1 and Table 2).

### 3.1. GRIN1

**Figure 2:**
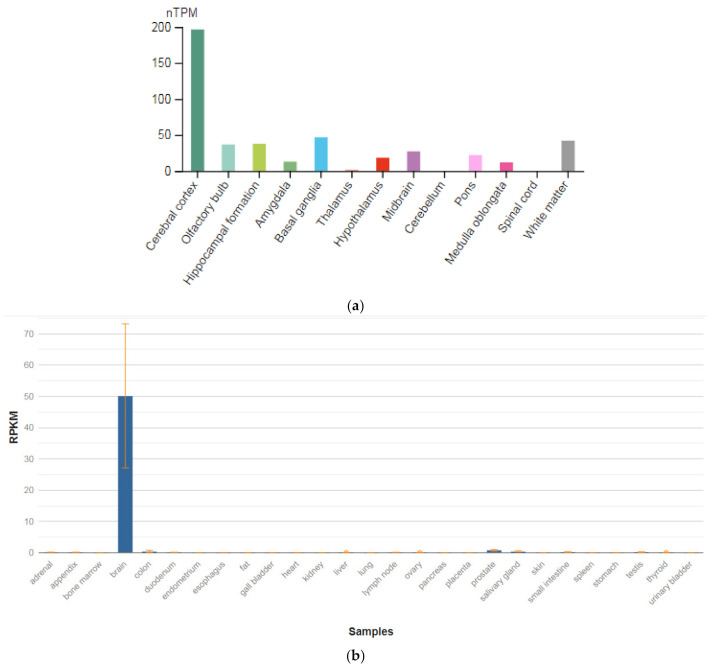
The tissue expression of the glutamate ionotropic receptor NMDA type subunit 1 in the brain (**a**) and other organs in the human body (**b**).

The *GRIN1* gene is localized on chromosome 9q34.3. The protein encoded by this gene is a critical subunit of NMDAR, a member of the glutamate receptor channel superfamily, which are heteromeric protein complexes with multiple subunits organized to form a ligand-dependent ion channel, including two zeta subunits (GRIN1) and two epsilon subunits (GRIN2A, GRIN2B, GRIN2C, or GRIN2D). GRIN1 subunits play a key role in the plasticity of synapses in the central nervous system (CNS), which is believed to underlie memory and learning. Receptors are predominantly expressed in the brain (Figure 2a,b) [47]. Cell-specific factors control the expression of various isoforms, possibly contributing to the functional diversity of subunits. Alternatively spliced transcript variants are described.

*GRIN1* mRNA is expressed in neuron-enriched regions of the developing and adult brains. These results demonstrate that GRIN1 is a neuron-specific protein. Together with neuronal specific expression and dendritic/axonal localization of the GRIN1 protein, it is assumed that GRIN1 plays an important role in the differentiation and arrangement of neuronal cells [48]. Impairment of NMDAR signaling within GABAergic interneurons is emerging to be a key driver of juvenile-onset neural circuit disorders [49]. In particular, early postnatal ablation of the obligate NMDAR subunit gene GRIN1 in GABAergic interneurons [50,51,52] resembles global GRIN1 loss in the constellation of schizophrenia-like behavioral and neural circuit aberrations [53].

Abnormal glutamate ionotropic NMDAR type subunit 1 (NR1) may be a potential cause of schizophrenia. Liu et al. [54] conducted a case–control study to investigate the association between the *GRIN1* gene, which encodes the NR1 subunit, and the risk of schizophrenia in a northern Chinese Han population. Seven SNVs (rs112421622 (-2019 T/C), rs138961287 (-1962–1961insT), rs117783907 (-1945G/T), rs181682830 (-1934G/A), rs7032504 (-1742C/T), rs144123109 (-1140G/A), and rs11146020 (-855 G/C)) were detected in the study population. Rs117783907 (-1945G/T) was associated with the occurrence of schizophrenia as a protective factor. The genotype frequencies of rs138961287 (-1962–1961insT) and rs11146020 (-855G/C) were statistically different between cases and controls (*p* < 0.0083). The other four SNVs were not shown to be associated with schizophrenia. Two haplotypes were composed of the seven SNVs, and the distribution of T–del–G–G–C–G–G was significantly different between the case and control groups.

Georgia et al. [42] also considered the GRIN1 gene as a candidate gene for schizophrenia. The authors investigated the association of four SNVs (rs4880213, rs11146020, rs6293, and rs10747050) and one microsatellite marker (rs11146020) of the *GRIN1* gene with the risk of schizophrenia in the German population of European origin. A statistically significant association of one marker rs11146020 (heterozygous and homozygous genotypes G/C and C/C) was found. The significance was more pronounced in the subgroup of patients with schizophrenia who had a history of an episode of major depression.

In addition, SNVs in this gene can be considered as biomarkers of negative symptoms and cognitive disorders in patients with schizophrenia [55,56]. Interestingly, intergenic interaction in patients with folate deficiency can play an important role in the development of schizophrenia and comorbid depressive disorders, which is illustrated by the example of patients with schizophrenia in the Iranian population [57] and on the NMDAR-knockout model of schizophrenia [51].

### 3.2. GPM6A

**Figure 3:**
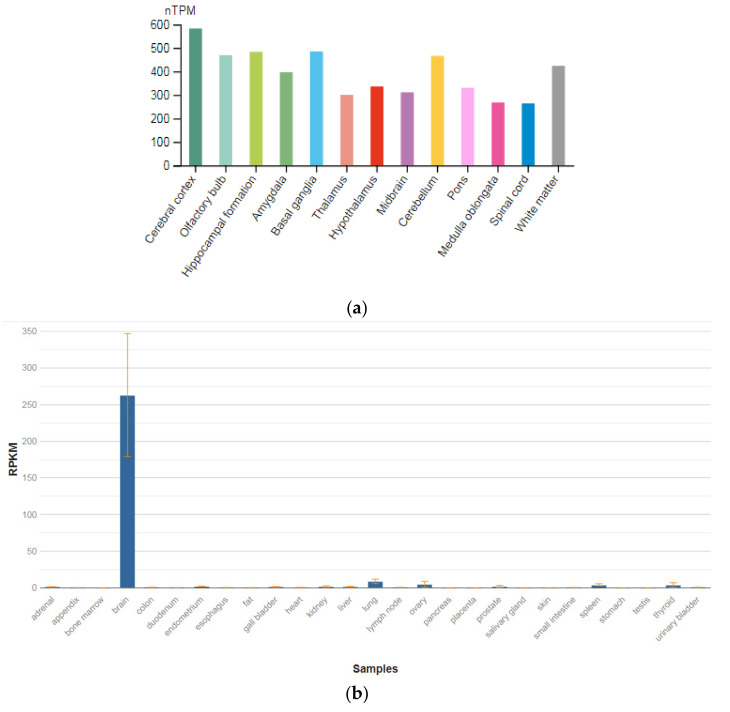
The tissue expression of the protein M6A in the brain (**a**) and other organs in the human body (**b**).

The *GPM6A* gene is localized on chromosome 4q34.2. The protein encoded by this gene is the neuronal membrane glycoprotein M6-A (Figure 3a). This protein participates in the differentiation of neurons, including the differentiation and migration of neuronal stem cells; it also plays a role in the plasticity of neurons and participates in the growth of neurites and filopodia, the mobility of filopodia, and possibly the formation of synapses. Moreover, it may participate in the neuronal NGF-dependent influx of Ca^2+^ ions, as well as in the regulation of endocytosis and intracellular transfer of G-protein-coupled receptors (GPCR). Lastly, it enhances internalization and recycling of opioid receptors mu-type. This protein is predominantly expressed in the brain (Figure 3b) [47].

The identification of signaling pathways involved in the development of psychiatric disorders aids the screening of possible therapeutic targets. SNVs in the *GPM6A* gene or alterations in GPM6A expression are linked to neurological disorders such as schizophrenia, depression, and Alzheimer’s disease. The neuronal surface glycoprotein M6a promotes filopodia/spines and dendrites [58]. A substantial body of evidence suggests that the extracellular loops of M6a command its function. However, the proteins that associate with them and that modulate neuronal plasticity have not yet been determined. Furthermore, endogenous M6a interacts with piccolo, synaptic vesicle protein 2B, and synapsin 1 in mature cultured hippocampal neurons [59].

Results of the study by Fuchsova et al. [60] indicated transcriptional downregulation of GPM6A and GPM6B in the hippocampus of depressed suicides. The expression level of calcium/calmodulin-dependent protein kinase II α (CAMK2A) and coronin1A (CORO1A) was also significantly decreased.

Ma et al. [61] systematically predicted the plausible candidate causal genes for schizophrenia at a genome-wide level. The authors utilized different approaches and strategies to predict causal genes for schizophrenia, including Sherlock, SMR, DAPPLE, Prix Fixe, NetWAS, and DEPICT. By integrating the results from different prediction approaches, they identified six top candidates that represent promising causal genes for schizophrenia (*CNTN4*, *GATAD2A*, *GPM6A*, *MMP16*, *PSMA4*, and *TCF4*), including the *GPM6A* gene.

Boks et al. [43] investigated the association of SNVs in the *GPM6A* gene with depressive disorder in patients with schizophrenia. The authors found a statistically significant association in a subgroup of patients with schizophrenia with a high level of depression (adjusted *p* = 0.006). Moreover, they showed the role of the *GPM6A* gene in modulating the effect of stress on the hippocampus on the example of an animal model of schizophrenia, which suggests that the *GMP6A* gene plays a role in stress-induced changes in the hippocampus, which are detected in mental disorders in general and schizophrenia in particular.

M6a is involved in neuron development and synapse formation and plasticity [62], and it was also recently proposed as a gene target in various neuropsychiatric disorders where it could also be used as a biomarker [63] of schizophrenia and depression disorder comorbidity.

### 3.3. SEPTIN4

**Figure 4:**
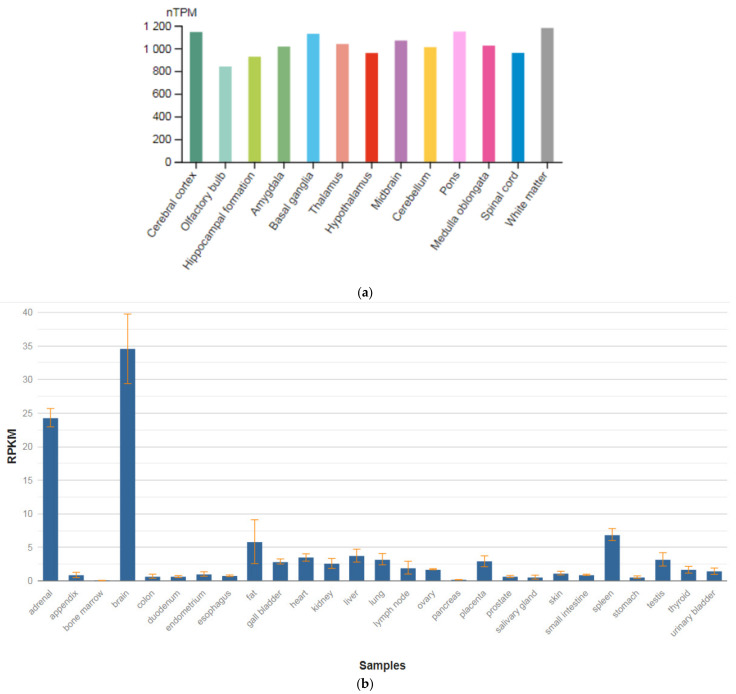
The tissue expression of the septin type 4 in the brain (**a**) and other organs in the human body (**b**).

The *SEPTIN4* (*SEPT4*) gene is localized on chromosome 17q22. This gene is a member of the septin family of nucleotide-binding proteins, originally described in yeast as proteins that regulate the cycle of cell division. Disruption of septic tank function 4 disrupts cytokinesis and leads to the formation of large multinucleated or polyploid cells. The gene itself is highly expressed in the brain, adrenal glands, and heart (Figure 4a,b) [47]. Alternatively, spliced transcript variants encoding different isoforms of the protein septin 4 (SEPT4) have been described for the *SEPTIN4* gene.

Schizophrenic brains exhibit various neuropathological changes in size, volume, and structure as compared to normal brains. These structural abnormalities could be the result of apoptotic cell death. SEPT4 plays an important role in the induction and promotion of apoptosis. Although SEPT4 is highly expressed in the healthy human brain, most tested schizophrenic brain samples showed no expression of SEPT4. Specifically, using Western blot analysis with monoclonal anti-SEPT4 antibody, Gottfried et al. [64] found that only one out of 14 schizophrenic samples (7%) showed a strong SEPT4 signal as compared to 10 out of 15 (66.6%) found in the normal control group. A fourfold reduction in apoptosis rate was measured in these schizophrenic samples as compared to the average apoptosis rate found in all other samples. These data support the linkage between loss of SEPT4 expression and the loss of sensitivity toward apoptosis. Interestingly, levels of SEPT4 were significantly lower in male schizophrenic patients as compared to female schizophrenic patients and males of all other control groups. This study proposed that the *SEPTIN4* gene may play an important role in the pathogenesis of schizophrenia and could be used as a biomarker for this disease.

Boks et al. [43] showed an association of the SNVs in the *SEPTIN4* gene in schizophrenic patients with primary drug resistance. However, the role of this gene in the development of comorbid depressive disorders in schizophrenic patients needs clarification.

### 3.4. TPH1

**Figure 5:**
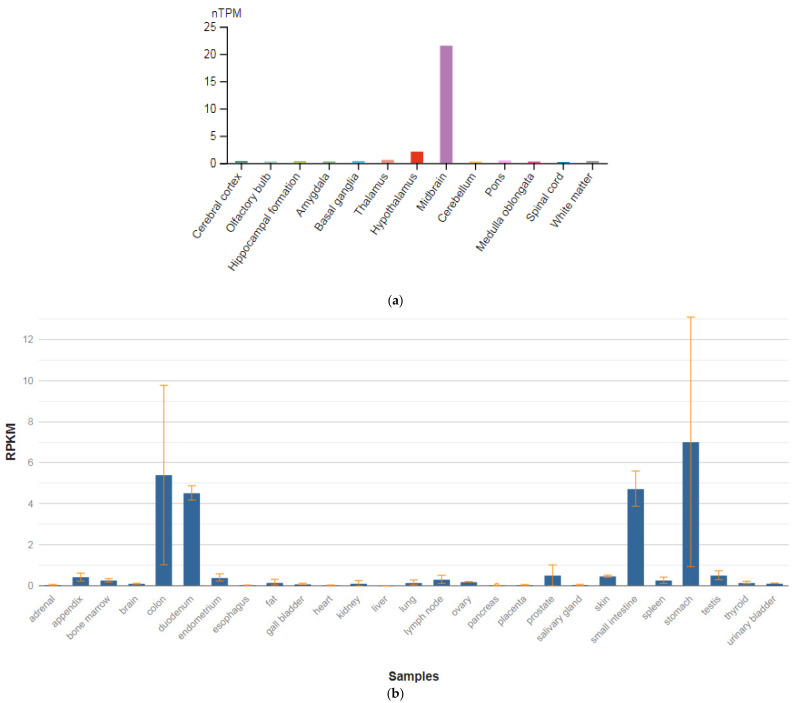
The tissue expression of tryptophan hydroxylase type 1 in the brain (**a**) and other organs in the human body (**b**).

The *TPH1* gene is localized on chromosome 11p15.1. This gene encodes an enzyme, a member of the aromatic amino-acid hydroxylase family, tryptophan hydroxylase type 1 (TPH1) (Figure 5a,b), which is widely expressed in various organs and tissues in humans [47]. The enzyme TPH1 catalyzes the first and rate-limiting stage of the biosynthesis of serotonin, an important hormone and neurotransmitter. Mutations and SNVs / polymorphisms in this gene are associated with an increased risk of various diseases and disorders, including schizophrenia, anxiety disorders with somatic manifestations, depression, bipolar disorder, suicidal behavior, and addiction.

Allen et al. [65] performed a meta-analysis across all ancestries comparing 829 patients with schizophrenia with 1268 controls and found that the A versus C allele at position 218 in intron 7 (rs1800532) of the *TPH1* gene was associated with susceptibility to schizophrenia (OR, 1.31; 95% CI, 1.15–1.51; *p*-value = 0.0008). According to the Venice guidelines for the assessment of cumulative evidence in genetic association studies [66], SNV in the *TPH1* gene association showed a ‘strong’ degree of epidemiologic credibility.

Li and He [67] found an overall association between depressive disorders with suicidal behavior and the *TPH* A779C/A218C polymorphisms in a meta-analysis of 34 case–control studies from 21 published articles and an unpublished paper. The A allele was the risk allele for both polymorphisms, yielding an odds ratio of 1.12 overall for carriers of both A alleles. Sand [68] commented that the report of Li and He [67] used pooled populations, pooled SNVs frequencies, imprecise phenotypes, and redundant reports, and he questioned the finding of allelic association.

Abbar et al. [69] investigated the role of the *TPH1* gene as a predictor of depressive disorders and suicidal behavior among patients with schizophrenia. The authors investigated seven SNVs among patients of European origin with suicidal behavior compared with a control group of similar age. A statistically significant association with suicidal behavior was shown for SNVs located in introns 7, 8, and 9 (*p*-value = 0.0008). These loci were in complete nonequilibrium coupling. In addition, a statistically significant association was also demonstrated in 39 noncoding regions of the *TPH1* gene (*p*-value = 0.0014). This association was strongest in patients who attempted suicide by violent methods and who had a history of major depression. However, no statistically significant association was found between the SNVs located in the promoter region, intron 1 and intron 3 of the *TPH1* gene, and suicidal attempts.

### 3.5. TPH2

**Figure 6:**
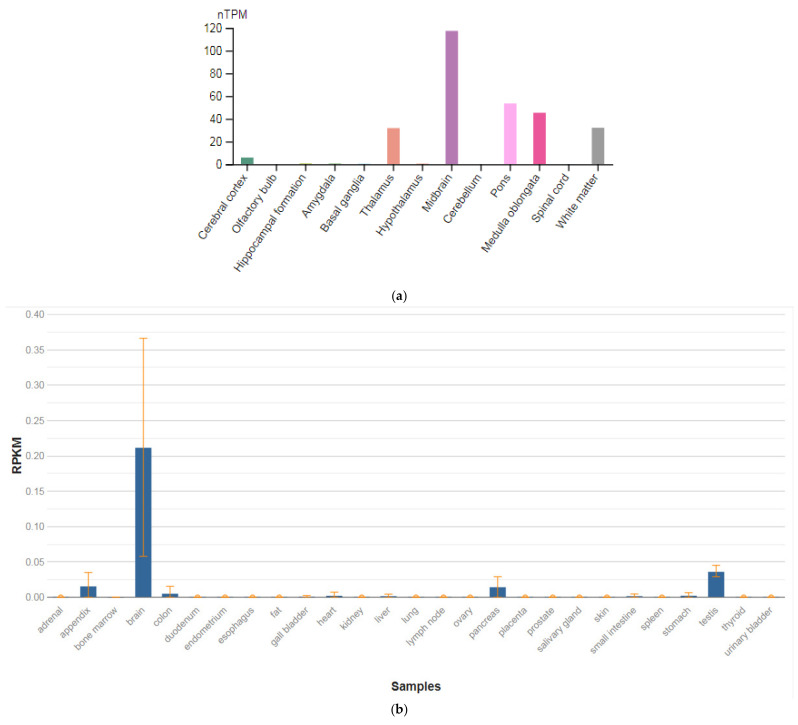
The tissue expression of the tryptophan hydroxylase type 2 in the brain (**a**) and other organs in the human body (**b**).

The *TPH2* gene is localized on chromosome 12q21.1. This gene encodes an enzyme, a member of the family of pterin-dependent aromatic amino-acid hydroxylases, tryptophan hydroxylase type 2 (TPH2). TPH2 is the rate-limiting enzyme in the synthesis of serotonin (5-hydroxytryptamine, or 5HT). 5HT is causally involved in numerous CNS activities (Figure 6a), and it has several functions in peripheral tissues, including the maintenance of vascular tone and gut motility (Figure 5b) [47]. Mutations in this gene may be associated with psychiatric disorders such as bipolar affective disorder, severe unipolar depression, and schizophrenia. Moreover, using brain functional magnetic resonance imaging (fMRI), Brown et al. [70] found that SNV 844G > T in the upstream regulatory region of the *TPH2* gene biased the reactivity of the amygdala, suggesting that the T allele may be associated with greater promoter activity.

Cichon et al. [71] found an association between a 757C–T transition in exon 6 of the *TPH2* gene, resulting in a pro206-to-ser (P206S) substitution (rs17110563) and bipolar affective disorder (125480) in two cohorts from Germany and Russia (883 patients and 1300 controls). In the combined sample, the ser206 allele yielded (OR = 4.8) for development of bipolar affective disorder (*p* = 0.0024). In vitro functional expression studies showed that the P206S protein had similar catalytic activity as wildtype, but decreased solubility and thermal stability. Further studies suggested a mild dominant negative effect.

However, Garriock et al. [72] found no evidence of R441H mutation of the *TPH2* gene by sequence analysis of 182 patients with unipolar depression (83 were treatment-resistant), 186 nondepressed controls, and eight bipolar patients. The ethnicity and gender distributions were similar to those studied by Zhang et al. [73].

Bellivier and Chaste [74] conducted associative studies of the role of the SNVs of the gene *TPH2* with suicidal behavior among patients suffering from schizophrenia. Special attention was paid by the authors to SNV 218A > C of the *TPH2* gene. The OR for suicidal behavior in the study sample in carriers of the major A allele with suicidal behavior was 1.62 (95% CI = 1.26—2.07). In addition, the authors conducted a meta-analysis of associative studies conducted earlier, which also showed a statistically significant association between the SNV 218A > C of the *TPH2* gene and suicidal behavior. Both the original study and the results of the meta-analysis indicated that the carriage of the A allele has a dose-dependent effect (homozygote > heterozygote) on the risk of suicidal behavior, but not depressive disorders.

Zhang et al. [75] identified a glutamine-to-alanine transition at nucleotide 1463 of the *TPH2* gene that replaces the highly conserved arginine at position 441 with histidine (R441H). This mutation resulted in an approximately 80% loss of function in serotonin production when *TPH2* was expressed in PC12 cells. SNV analysis in a cohort of 87 patients with unipolar major depression revealed that nine patients carried the mutant allele, while, among 219 controls, three subjects carried this mutation. Interestingly, depressed patients with this mutation were poorly responsive or unresponsive to SSRIs. The three control subjects with this mutation were not diagnosed as having unipolar major depression, but they displayed clinical symptoms of comorbid conditions. One of the three control subjects with homozygous mutant alleles had generalized anxiety symptoms, while the other two with heterozygous alleles had mild depression and a family history of mental illness or drug and alcohol abuse, suggesting a potentially higher susceptibility for certain neuropsychiatric disorders in the presence of the mutant allele. This mutation was not identified in 60 patients with bipolar disorder.

Garriock et al. [76], however, found no evidence of R441H mutation of the *TPH2* gene by sequence analysis of 182 patients with unipolar depression (83 were treatment-resistant), 186 nondepressed controls, and eight bipolar patients. The ethnicity and gender distributions were similar to those studied by Zhang et al. [75].

### 3.6. CACNA1C

**Figure 7:**
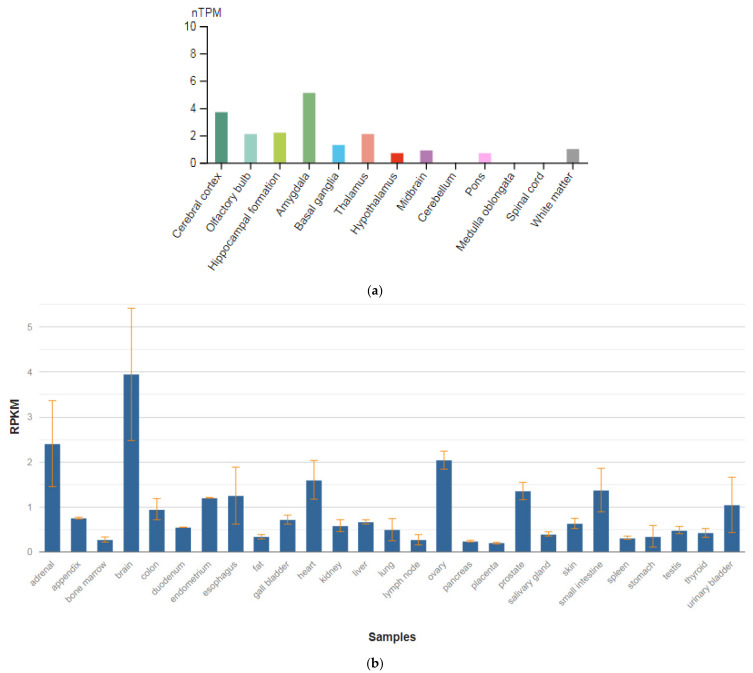
The tissue expression of calcium voltage-gated channel subunit alpha1 C in the brain (**a**) and other organs in the human body (**b**).

The *CACNA1C* gene is localized on chromosome 12p13.33. This gene encodes the α-1 subunit of the potential-dependent calcium channel. Calcium channels mediate the influx of calcium ions into the cell during membrane depolarization. The α-1 subunit consists of 24 transmembrane segments and forms pores through which ions pass into the cell. The calcium channel consists of a complex of α-1, α-2/delta, β, and γ subunits in a ratio of 1:1:1:1. There are many isoforms of each of these proteins, which are either encoded by different genes or are the result of alternative splicing of transcripts. The protein encoded by this gene is bound to dihydropyridines and is inhibited by them. Alternative splicing leads to the appearance of many transcript variants encoding different proteins. Among the associated pathways is the transmission of the adenosine diphosphate (ADP) signal through the purinoceptor P2Y12 and the integrin pathway. These receptors are expressed in various organs and tissues in humans (Figure 7b), but the highest level of expression is observed in the brain (Figure 7a) [47].

Smoller et al. [41] studied the SNVs in the genes *CACNA1C* and *CACNB2* encoding two subunits of potential-dependent calcium channels on chromosomes 3p21 and 10q24, respectively. The authors showed the relationship of comorbid disorders (schizophrenia and depression), especially with adult onset, compared with childhood onset. The authors suggested that SNVs in the studied genes affect the activity of L-type calcium channels of the CNS, which, in turn, has a pleiotropic effect on the mental condition.

### 3.7. CACNB2

**Figure 8:**
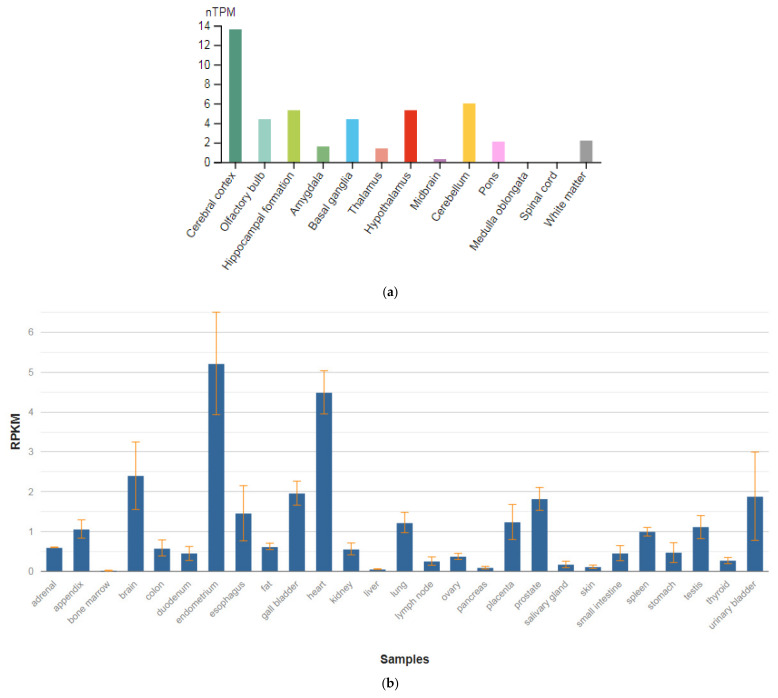
The tissue expression of the calcium voltage-gated channel auxiliary subunit β 2 in the brain (**a**) and other organs in the human body (**b**).

The *CACNB2* gene is localized on chromosome 10p12.31. This gene encodes a subunit of the potential-dependent calcium channel protein, which is a member of the superfamily of potential-dependent calcium channels. The gene product was initially identified as an antigenic target in Lambert–Eaton myasthenic syndrome (autoimmune neuromuscular disease). It was later shown that mutations in this gene are associated with Brugada syndrome. Among the pathways associated with this receptor, the integrin pathway and the integration of energy metabolism are important. Gene ontology annotations (GO) related to this gene include the activity of calcium channels and the activity of calcium channels controlled by high voltage. Williams et al. [76] isolated a cDNA corresponding to the β-2 subunit of a voltage-dependent calcium channel from a human hippocampus cDNA library. The deduced 478 amino acid protein has a calculated molecular mass of 53 kD. Their evidence was for various tissue-specific transcripts in the brain (Figure 8a), skeletal muscle, aorta, and other tissue and organ in human body (Figure 8b) [47].

The Cross-Disorder Group of the Psychiatric Genomics Consortium [41] analyzed genome-wide SNVs data for five disorders (autism spectrum disorder, attention deficit-hyperactivity disorder, bipolar disorder, major depressive disorder, and schizophrenia) in 33,332 cases and 27,888 controls of European ancestry. Characterizing allelic effects on each disorder, the authors applied a multinomial logistic regression procedure with model selection to identify the best-fitting model of relations between genotype and phenotype. They examined cross-disorder effects of genome-wide significant loci previously identified for bipolar disorder and schizophrenia, and they used polygenic risk-score analysis to examine such effects from a broader set of common variants. The authors used enrichment analysis of expression quantitative trait loci (eQTL) data to assess whether SNVs with cross-disorder association were enriched for regulatory SNVs in postmortem brain tissue samples. SNVs at four loci surpassed the cutoff for genome-wide significance (*p* < 0.000000005) in the primary analysis: regions on chromosomes 3p21 and 10q24, and SNVs in the *CACNA1C* and *CACNB2* genes. Model selection analysis supported effects of these loci for several disorders. Loci previously associated with bipolar disorder or schizophrenia had variable diagnostic specificity. Polygenic risk scores showed cross-disorder associations, notably with adult-onset disorders. Pathway analysis supported a role for calcium channel signaling genes for all five disorders. Lastly, SNVs with evidence of cross-disorder association were enriched for brain eQTL markers.

In the study by Juraeva et al. [77], an integrated hierarchical approach was applied to identify pathways associated with susceptibility to schizophrenia, detect genes that may be potentially affected in these pathways since they contain an associated polymorphism, and annotate the functional consequences of such SNVs in the affected genes or their regulatory regions. The global test was applied to detect schizophrenia-associated pathways using discovery and replication datasets comprising 5040 and 5082 individuals of European ancestry, respectively. Information concerning functional gene sets was retrieved from the Kyoto Encyclopedia of Genes and Genomes, Gene Ontology, and the Molecular Signatures Database. Fourteen of the gene sets or pathways identified in the discovery dataset were confirmed in the replication dataset. For two genes, i.e., *CTCF* and *CACNB2*, evidence for association with schizophrenia was available (at the gene level) in both the discovery study and the published data from the Psychiatric Genomics Consortium schizophrenia study. Furthermore, these genes mapped to four of the 14 presently identified pathways. Several of the SNVs assigned to *CTCF* and *CACNB2* have potential functional consequences, and a gene in close proximity to *CACNB2*, i.e., *ARL5B*, was identified as a potential gene of interest in schizophrenia and depressive disorder comorbidity.

Additionally, Smoller et al. [41] showed the role of the SNVs in the *CACNB2* gene in the development of a number of psychiatric disorders with childhood onset, including schizophrenia and bipolar disorder. These results provide evidence relevant to the goal of going beyond descriptive syndromes in psychiatry to nosology based on the causes of the disease.

### 3.8. BCL9

**Figure 9:**
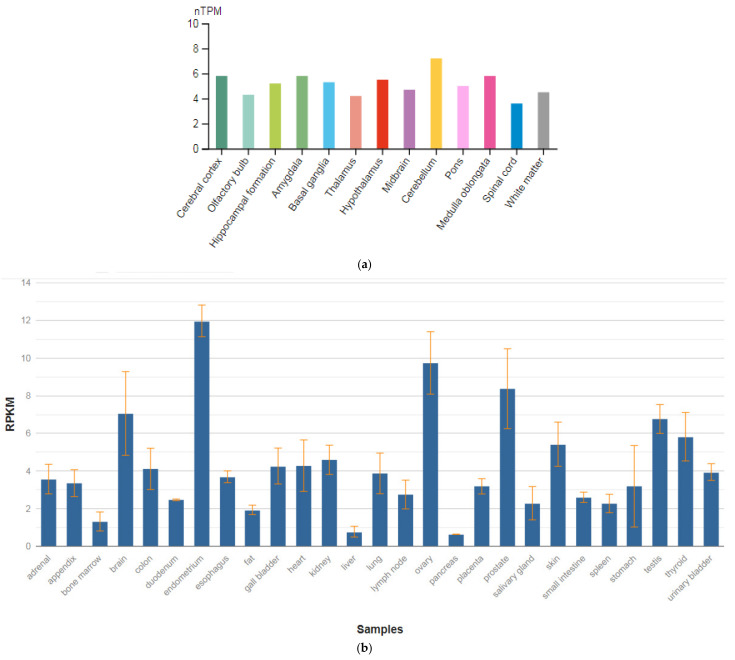
The tissue expression of the BCL6 transcription repressor in the brain (**a**) and other organs in the human body (**b**).

The *BCL9* gene is located on chromosome 1q21.1. Its function is unknown. Overexpression of BCL9 may have pathogenic significance in malignant neoplasms of B cells. BCL9 (BCL9 transcription coactivator) is a gene encoding a protein of the same name. Among the associated pathways is the regulation of Wnt-mediated β-catenin signaling and transcription of the target gene and signaling by GPCR. This protein is expressed in the brain (Figure 9a), as well as various organs and tissues in the human body (Figure 9b) [47].

Recent genome-wide association studies revealed that common variations and rare copy-number variations contribute to the risk of mental disorders. Rare recurrent microdeletions at 1q21.1 were reported to be associated with schizophrenia, and the *BCL9* gene at 1q21.1 was also a functional candidate gene for mental disorders. Thus, by using cumulative scoring, Luo et al. [78] systematically prioritized the genes affected by SNVs in schizophrenia. The authors identified eight top genes that are frequently disrupted by SNVs, including the *BCL9* gene.

Li et al. [79] conducted a three-stage case–control study in Shanghai, China. A total of 12,229 subjects were included: 5772 normal controls, 4187 patients with schizophrenia, 1135 patients with bipolar disorder, and 1135 patients with major depressive disorder. To determine main outcome measures during the first and second stages of this study, the authors genotyped 10 SNVs using the ligation detection reaction method. During the third stage of the study, all SNSs were genotyped using TaqMan technology. The first stage demonstrated that rs672607 was significantly associated with schizophrenia (*p* = *p*-value 0.0000269). During the second stage, rs672607 was successfully replicated (*p*-value = 0.0000133), while rs9326555 (*p*-value = 0.002), rs1240083 (*p*-value = 0.00017), and rs688325 (*p*-value = 0.006) were newly identified to be significant. During the third stage, all SNVs were genotyped in 1135 patients with schizophrenia, 1135 patients with bipolar disorder, 1135 patients with major depressive disorder, and 1135 normal controls for further validation. When the authors combined all the data from the three stages, they found that rs9326555 (*p*-value = 0.0000153), rs10494251 (*p*-value = 0.02), rs1240083 (*p*-value = 0.000152), rs672607 (*p*-value = 0.0000000000123), rs688325 (*p*-value = 0.000254), and rs3766512 (*p*-value = 0.01) were significant. One SNV (rs672607) was significant in major depressive disorder (*p*-value = 0.001) and bipolar disorder (*p*-value = 0.03), while rs10494251 (*p*-value = 0.04), rs1541187 (*p*-value = 0.04), rs688325 (*p*-value = 0.02), and rs946903 (*p*-value = 0.006) were significant in major depressive disorder. This study demonstrated that common SNVs in the *BCL9* gene confer risk of schizophrenia and may also be associated with bipolar disorder and major depressive disorder in the Chinese Han population.

Negative symptoms in schizophrenia are attractive intermediate phenotypes according to their clinical and treatment response features. Therefore, Xu et al. [80] studied SNVs underlying the negative symptoms of schizophrenia by analyzing two genome-wide association datasets consisting of a total of 1774 European-American patients and 2726 controls. Logistic regression analysis of negative symptoms as a binary trait (adjusted for age and sex) was performed using PLINK. For meta-analysis of two datasets, the fixed-effect model in PLINK was applied. Through meta-analysis, the authors identified 25 SNV associated with negative symptoms with *p* < 0.00005. In particular, they detected five SNVs in the first two genes/loci strongly associated with negative symptoms of schizophrenia (*p*-value (meta-analysis) < 0.00000622), which included three SNVs in the *BCL9* gene: rs583583 showed the strongest association (*p*-value (meta-analysis) = 0.0000006) and two SNVs in the C9orf5 (the top SNV is rs643410, *p*-value = 0.00000129). This report revealed the common SNVs in the *BCL9* gene influencing negative symptoms of schizophrenia. These results provide direct evidence of using negative symptoms as an intermediate phenotype to dissect the complex genetics of schizophrenia. However, additional studies are warranted to examine the underlying mechanisms of these disease-associated SNVs in the *BCL9* gene.

A recent study reported that SNV rs583583 in the *BCL9* gene is associated with negative symptoms of schizophrenia, as measured by the Positive and Negative Syndrome Scale (PANSS), in the Caucasian population. Kimura et al. [81] investigated the genetic association of rs583583 with its effect on negative symptoms in the Japanese patients. For association analysis, the authors used a Japanese sample set comprising 1089 patients with schizophrenia and 950 controls. Analysis of the effect of rs586586 on negative symptoms as examined by PANSS was investigated using 280 patients with schizophrenia. Furthermore, for analysis of cognitive performance, they investigated 90 patients with schizophrenia and 51 controls using the Continuous Performance Test (CPT-IP) and the Wisconsin Card Sorting Test (WCST) Keio version. The authors did not detect an association between rs583583 and schizophrenia. Furthermore, rs583583 was not associated with PANSS negative scores or with CPT-IT or WCST cognitive tests. Considering the results of their previous study, combined with the results of the subsequent study of rs583583, the authors argued that the *BCL9* gene most likely does not harbor a common genetic variant that can increase the risk for schizophrenia (and negative symptoms of this disease) in the Japanese population.

## 4. Discussion

The cellular and molecular mechanisms underlying mental disorders show that most of them can be categorized as synaptopathies or damage of synaptic function and plasticity. Synaptic formation and maintenance are orchestrated by protein complexes that are in turn regulated in space and time during neuronal development allowing synaptic plasticity [63]. In addition, neurotransmission disorder plays an important role. However, the exact mechanisms via which these processes are managed remain unknown. Large-scale genomic and proteomic projects have led to the discovery of new molecules and their associated variants as disease risk factors.

Schizophrenia and depression are devastating mental illnesses that contribute substantially to the global burden of disease [1,3]. Moreover, schizophrenia patients have an elevated risk for developing depressive symptoms compared to the already high lifetime prevalence of depression in the general population [4]. Depression has been reported during all stages of the course of schizophrenia [5], and depressive symptoms are associated with an increased risk of suicide [9]. Methodological differences in diagnosis and time course of evaluation mean that there is a wide variance of depressive symptoms reported by patients with schizophrenia in the literature, with prevalence rates as high as 61% [12]. Nevertheless, reviews of the literature convincingly show that depression is elevated in schizophrenia [13].

Thus, patients with schizophrenia are at an increased risk for the development of depression. Overlap in the symptoms and genetic predictors between these disorders suggests that a common etiological mechanism may underlie the presentation of comorbid depression in schizophrenia. Understanding these shared mechanisms will be important in informing the development of new treatments [82].

The analysis of previously conducted genome-wide and associative genetic studies of comorbid conditions (schizophrenia and depressive disorders) indicates the similarity of genetic predictors and molecular mechanisms of the mental disorders under consideration, which correlates with epidemiological and clinical studies of the comorbidity of schizophrenia and depression. In addition, Hamshere et al. [44] investigated the comorbidity of schizophrenia with episodes of major depression. The authors examined microsatellite markers on two chromosomes at loci 4q28.3 and 20q11.21. The authors identified loci where candidate genes may be located, which play a role in predisposition to the development of episodes of major depression in patients suffering from schizophrenia.

From the position of rapidly developing personalized psychiatry [83], the translation of the results of genetic studies into clinical psychiatry is important because a personalized approach can predict the risk of depressive disorders in patients with schizophrenia, which can increase the effectiveness and safety of antipsychotic therapy, optimize the selection of antidepressants [84], improve the quality of life of patients with schizophrenia, and reduce the number of suicide attempts and completed suicides in the category of patients under consideration.

The majority of studies that can be considered clinically significant were devoted to the role of the following candidate genes: *GRIN1*, *GPM6A*, *TPH1*, *CACNA1C*, *CACNB*, and *BCL9* (Table 3 and Table 4). In addition, there has been an increase in the number of associative studies of SNVs and microsatellite genetic markers, which can encourage researchers and clinicians to consider the need to develop genetic panels and decision-making algorithms for their application in real clinical practice.

However, some studies included small sample sizes or were only studies of candidate genes, which may limit the reliability of the results and their translation into real clinical practice.

## 5. Limitations

The limitation of our topic review was the 10 years period of publication analysis. An additional restriction was searching for publications only in English and Russian. The quality of a few Russian publications has been criticized [85]. We removed these publications from the systemic analysis, thus limiting the publications to those in English as being more reliable (see Table 3 and Table 4). Furthermore, we did not analyze preprints or conference materials (for example, posters).

In addition, genetic studies on animal models of schizophrenia were not included in this thematic review, since studies of comorbid conditions (for example, schizophrenia and depression) cannot always be broadcast in animals and humans, although some studies in animals may undoubtedly be of scientific interest. Compared to mice models with classical depression or to schizophrenia models, animal models with schizophrenia and depression comorbidity present worse psychotic and depressive symptoms. These behavioral deficits are associated with impaired neuronal calcium activities in the frontal cortex and thalamic nuclei. Moreover, in sharp contrast to classical models that have a satisfactory response to antipsychotic or antidepressant drugs, this novel schizophrenia with depression model is resilient to combined drug treatment in terms of behavioral and functional recovery. Taken together, these data indicate that schizophrenia with depression likely involves a unique pathophysiology that is different from schizophrenia or depression alone [86]. Rodent models are powerful tools for understanding gene function as it relates to behavior. Examining rodent models relevant to both schizophrenia and depression reveals a number of common mechanisms. Current models which demonstrate endophenotypes of both schizophrenia and depression were reviewed here, including models of CUB and SUSHI multiple domains 1, PDZ and LIM domain 5, glutamate delta 1 receptor, diabetic db/db mice, neuropeptide Y, disrupted in schizophrenia 1 and its interacting partners, reelin, maternal immune activation, and social isolation. Neurotransmission, brain connectivity, the immune system, the environment, and metabolism emerge as potential common mechanisms linking these models and potentially explaining comorbid depression in schizophrenia [82]. As more models are discovered, the emerging picture of the shared pathophysiological mechanisms between schizophrenia and depression will become increasingly coherent. Interrogating precise molecular and neural substrates as they relate to specific endophenotypes, and carefully examining ‘gene × gene’ and ‘gene × environment’ interactions will contribute to a better understanding of the neurobiological mechanisms of comorbidity in mental illness. This understanding will inform future efforts in developing treatments for neuropsychiatric comorbidity [82]. Future research should then focus on differentiating precise mechanisms and their relationships to these highly integrated systems. Advancing technologies such as optogenetics and light sheet microscopy should aid in deciphering the roles of specific neural circuitry [87].

An interesting question is the influence of gender, ethnicity, and race of patients on the realization of genetic predisposition in the pathological phenotype of comorbidity of schizophrenia and depression. On the one hand, our earlier meta-analysis of the incidence of comorbidity of schizophrenia and depression in the world did not demonstrate statistically significant gender differences, although we showed a higher incidence of this phenotype in patients with schizophrenia in the Middle East, Southeast Asia, and Russia [13] On the other hand, it is difficult to assess the influence of patients’ age on the realization of a genetic predisposition to the development of this endophenotype (schizophrenia + depression), since not all the publications we analyzed provided sufficient data for subsequent statistical processing.

These topics are important to discuss in future reviews.

## 6. Conclusions

Currently, there is no doubt about the importance of studying the comorbidity of schizophrenia and depressive disorders from a scientific and practical point of view. Genetic predictors and molecular mechanisms of schizophrenia and depression may be the same in some patients. Planning and conducting genome-wide and associative genetic studies of the comorbid condition under consideration in psychiatry is important for identifying biological and clinical predictors and developing a personalized strategy for the treatment of schizophrenia. However, it should be recognized that the problems of predictive and personalized psychiatry in the field of diagnosis and treatment of schizophrenia and comorbid disorders (primarily depression) are far from being resolved.

## Figures and Tables

**Table 1:** Genes responsible for schizophrenia and depression and diseases caused by their mutations (adapted by the authors from [45,46]).

Gene: MIM (Protein)	Chromosome Location	Clinical Manifestations of Mutation: MIM	Inheritance
*GRIN1*: 138249 (Glutamate ionotropic receptor NMDA type subunit 1)	9q34.3	Neurodevelopmental disorder with or without hyperkinetic movements and seizures, autosomal dominant: 614254 Neurodevelopmental disorder with or without hyperkinetic movements and seizures, autosomal recessive: 617820 Schizophrenia spectrum disorder: 181500	AD AR Mu
*GPM6A*: 601275 (Glycoprotein M6A)	4q34.2	Schizophrenia spectrum disorder: 181500	Mu
*SEPTIN4 or SEPT4*: 603696 (Septin 4 or Apoptosis-related protein in the TGF-β signaling pathway)	17q22	Meckel syndrome, type 1: 249000 Schizophrenia spectrum disorder: 181500	AR Mu
*TPH1*: 191060 (Tryptophan hydroxylase type 1)	11p15.1	Schizophrenia spectrum disorder: 181500	Mu
*TPH2*: 607478 (Tryptophan hydroxylase type 2 or Neuronal tryptophan hydroxylase)	12q21.1	Susceptibility to attention deficit–hyperactivity disorder: 613003 Susceptibility to unipolar depression: 608516 Susceptibility to bipolar affective disorder: 125480 Schizophrenia spectrum disorder: 181500	Mu Mu Mu Mu
*CACNA1C*: 114205 (α-1C subunit of voltage-dependent calcium channel type L)	12p13.33	Brugada syndrome type 3: 611875 Long QT syndrome type 8: 618447 Timothy syndrome: 601005 Schizophrenia spectrum disorder: 181500	Mu Mu AD Mu
*CACNB2*: 600003 (β2 subunit of voltage-dependent calcium channel)	10p12.33–p12.31	Brugada syndrome type 4: 611876 Schizophrenia spectrum disorder: 181500	Mu Mu
*BCL9*: 602597 (B-cell of lymphoma type 9 transcription coactivator)	1q21.2	Schizophrenia spectrum disorder: 181500	Mu

**Table 2:** Genes responsible for schizophrenia and depressive disorder comorbidity and their expression in the human brain (adapted by the authors from [47]).

Gene (MIM)	Expression Level in Brain (RPKM)
*GRIN1* (138249)	50.139 ± 23.048
*GPM6A* (601275)	262.992 ± 83.861
*SEPTIN4* (603696)	34.56 ± 5.181
*TPH1* (191060)	0.102 ± 0.025
*TPH2* (607478)	0.212 ± 0.154
*CACNA1C* (114205)	2.4 ± 0.846
*CACNB2* (600003)	3.947 ± 1.468
*BCL9* (602597)	7.058 ± 2.222

**Table 3:** Candidate genes predisposing to comorbidity of schizophrenia and depression.

Gene	Chromosome (Locus)	Schizophrenia	Depression	Comorbidity of Schizophrenia and Depression
*GRIN1*	9q34.3	(+) [38]	(+) [38]	(+) [42]
*GPM6A*	4q34.2	(+) [39]	(+) [39]	(+) [43]
*SEPTIN4*	17q22	(+) [39]	(−) [39]	(−) [43]
*TPH1*	11p15.3-p14	(+) [43]	(+) [43]	(+) [73]
*TPH2*	12q21.1	(+) [42]	(+) [42]	(−) [69]
*CACNA1C*	12p13.33	(+) [45]	(+) [45]	(+) [41]
*CACNB2*	10p12.31	(+) [45]	(+) [45]	(+) [41]

Note: (+)—association found; (−)—association not found.

**Table 4:** Candidate genes and their single nucleotide variants associated with comorbidity of schizophrenia and depression.

Gene	Chromosome Location	Single Nucleotide Variant	Patients Nationality (Race)	References
*GRIN1*	9q34.3	rs4880213 rs11146020 rs6293 rs10747050	European (German)	[42]
*GPM6A*	4q34.2	rs9247 rs3925 rs2286435 rs8135641 rs7748777 rs2240432 rs4917450 rs6434387 rs2293759	Caucasians and Africans (Americans)	[73]
*TPH1*	11p15.3-p14	T1606C T3792A A218C T465C C160T distal region of exon 11 39 (repeat expansion 5657 base pairs (CT) m (CA) n (CT) p)	Caucasians and African Americans	[73]
*CACNA1C*	12p13.33	rs1024582	Europeans (Norwegians)	[41]
*CACNB2*	10p12.31	rs2799573	Europeans (Norwegians)	[41]
*BCL9*	1q21.1	rs672607	Asians (Chinese)	[79]

## Data Availability

Not applicable.
